# Mobility of Cellulose Nanocrystals in Porous Media: Effects of Ionic Strength, Iron Oxides, and Soil Colloids

**DOI:** 10.3390/nano10020348

**Published:** 2020-02-18

**Authors:** Shuang Xu, Chongyang Shen, Xueyong Zhang, Xijuan Chen, Mark Radosevich, Siqun Wang, Jie Zhuang

**Affiliations:** 1 Key Laboratory of Pollution Ecology and Environmental Engineering, Institute of Applied Ecology, Chinese Academy of Sciences, Shenyang 110016, China; xushuang.iae@hotmail.com (S.X.); chenxj@iae.ac.cn (X.C.); 2 College of Land and Environment, Shenyang Agricultural University, Shenyang 110866, China; 3 Department of Soil and Water Sciences, China Agricultural University, Beijing 100193, China; chongyang.shen@cau.edu.cn; 4 School of Environmental Science, Liaoning University, Shenyang 110036, China; zhangxueyong009@foxmail.com; 5 Department of Biosystems Engineering and Soil Science, Center for Environmental Biotechnology, University of Tennessee, Knoxville, TN 37996, USA; mrad@utk.edu; 6 Department of Forestry, Wildlife and Fisheries, The University of Tennessee, Knoxville, TN 37996, USA; swang@utk.edu

**Keywords:** nanoparticle transport, natural soil, iron oxide, solution chemistry, cellulose nanocrystals

## Abstract

Understanding the dispersivity and migration of cellulose nanocrystals (CNCs) in porous media is important for exploring their potential for soil and water remediation. In this study, a series of saturated column experiments were conducted to investigate the coupled effects of ionic strength, iron oxides (hematite), and soil colloids on the transport of CNCs through quartz sand and natural soils (red earth and brown earth). Results showed that CNCs had high mobility in oxide-free sand and that iron oxide coating reduced the mobility of CNCs. An analysis of Derjaguin-Landau-Verwey-Overbeek interactions indicated that CNCs exhibited a deep primary minimum, nonexistent maximum repulsion and secondary minimum on hematite-coated sand, favorable for the attachment of CNCs. The maximum effluent percentage of CNCs was 96% in natural soils at 5 mM, but this value decreased to 4% at 50 mM. Soil colloids facilitated the transport of CNCs in brown earth with larger effect at higher ionic strength. The ionic strength effect was larger in natural soils than sand and in red earth than brown earth. The study showed that CNCs can travel 0.2 m to 72 m in porous media, depending on soil properties, solution chemistry, and soil colloids.

## 1. Introduction

Nanoparticles have been intentionally added into contaminated soil for either immobilization or remobilization of contaminants (e.g., leaching) because of their high specific surface area and strong binding capability [1,2,3,4,5,6]. Nanoparticles may also be unintentionally released into the soil during manufacturing, transportation, consumption, and disposal [7,8]. There have been concerns on the health risk posed by nanoparticles and their potential influences on the ecosystems. For example, a number of studies have demonstrated that many engineered nanoparticles (such as nanoscale metal oxide and plastic particles) posed health implications to water and terrestrial biota when consumed above certain threshold concentration [9,10,11,12,13,14,15]. Such concerns gave momentum to the utilization of renewable, biodegradable, and environmentally friendly carbon-based nanomaterials.

Cellulose nanocrystals (CNCs) are attractive candidates for environmental remediation due to their relatively low cost, unlimited supply, biodegradability, surface functionalization capabilities [16,17,18,19] and low biological toxicity [20]. CNCs are produced from cellulose via an acid hydrolysis process that disrupts the hydrogen bonds and cleaves the amorphous domains of the fiber to yield well-defined crystalline rods [21]. The dimension of CNCs is 4–20 nm in diameter and 100–250 nm in length depending on the raw material and separation method [22,23,24]. The small size leads to a high aspect ratio and a large specific surface area of CNCs [25]. Moreover, the existence of numerous hydroxyl groups allows for the incorporation of chemical moieties [26,27,28,29,30]. In recent years, the use of CNCs in wastewater treatment applications has gained increasing attention [16,18,31,32,33,34]. It has been reported that CNCs are electrostatically stable and have high dispersivity in wastewater [35,36]. Furthermore, surface modification can significantly enhance the ability of CNCs to remove heavy metals from aqueous solutions [23,31,37]. Due to the strong adsorption of heavy metals, CNCs may also be useful for remediation of heavy metal contaminated soil (i.e., removing metal ions from soil solution) if they can be extensively distributed in the contaminated zone. However, the factors and mechanisms that control the mobility of CNCs in porous media remain unclear to date.

Natural soils are very different from model porous media (e.g., quartz sand) in terms of soil solution chemistry, metal oxides, and soil colloids. The mechanisms that these factors influence the transport of nanoparticles through porous media have been well-documented [38,39]. The retention of nanoparticles in secondary energy minima on soil surfaces is enhanced at higher ionic strength because the secondary minimum depth increases with increasing ionic strength [40,41]. The heterogeneously charged soils usually carry patches of iron oxides on their surfaces, and they can enhance the retention of nanoparticles due to strong electrostatic attraction [42,43]. Soil colloids are the most active constituent of soil, which refers to small soil particles with sizes from nanometers to a few micrometers and include crystalline or non-crystalline silicate clays, iron and aluminum oxides, and humus. Soil colloids are potential carriers of contaminants in the subsurface environment due to their large specific surface area, strong adsorption, high surface reactivity, and various charge characteristics. The interaction or association of soil colloids with nanoparticles is known to affect the fate of nanoparticles in soils. For instance, the migration of soil colloids can promote the transport of nanoparticles [44,45] and likely vice versa.

The objective of this study was to understand the transport behavior of CNCs in a model porous medium (quartz sand) and two natural loam soils by quantifying the impacts of ionic strength, iron oxides, and soil colloids. Breakthrough curves for CNCs were determined through saturated column experiments. A classic convection-dispersion equation (CDE) that includes a time- and depth-dependent deposition rate was employed to simulate the transport of CNCs. The Derjaguin-Landau-Verwey-Overbeek (DLVO) interaction energies were calculated to interpret the mechanisms controlling the attachment and transport of CNCs. The maximum travel distance was evaluated to estimate the mobility of CNCs in porous media. This study is the first to investigate transport of CNCs, and the findings have important implications for soil remediation or other applications with carbon-based nanomaterials.

## 2. Materials and Methods 

### 2.1. Porous Media, Soil Colloids, and CNCs 

**Figure 1:**
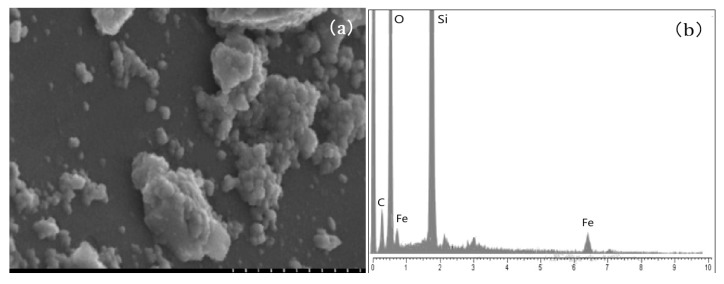
Scanning electron microscopy (**a**) and energy-dispersive X-ray analysis (**b**) of hematite-coated sand.

The transport experiments were performed using three porous media (quartz sand and two loamy soils). Quartz sand 20/40 (Sinopharm Chemical Reagent Co., Ltd., Shanghai, China), containing 99.2% of silicon dioxide and with a specific gravity of 2.7 g cm^−3^, was employed in the column experiments. Prior to use, the sand was dry-sieved to select the size fraction of 0.5–0.75 mm and rinsed with deionized water to get rid of suspended impurities. The sand was then ultrasonicated in 0.01 M NaOH solution for 30 min, rinsed with deionized water, and then ultrasonicated for another 30 min in 0.01 M HCl solution [46]. The sand was finally rinsed with deionized water until the electric conductivity of water became zero and dried in an oven at 105 °C, heretofore referred to as oxide-free sand. Preparation of hematite-coated sand was carried out according to the procedure described by Benjamin et al. [47]. Specifically, 80 mL of a 2.5 M FeCl_3_ solution was poured over 200 mL quartz sand, and the mixture was stirred hourly and heated at 110 °C for 3 h, by which time it appeared to be dry. The temperature was then raised to 550 °C for additional 3 h, and the sand was cooled in air. Upon rinsing, a dark red coating remained on the sand surface. Scanning electron microscopy (SEM, Regulus 8100, Hitachi, Japan) was performed to reveal the morphology of the hematite-coated sand (Figure 1a), and energy-dispersive X-ray analysis (EDXA) was used to confirm the presence of hematite on sand (Figure 1b). 

The two loamy soils, brown earth and red earth, were collected from the topsoil (0–20 cm) of a no-till farmland located in Shenyang Experimental Station of Agroecology (Shenyang, China; 41°31′ N, 123°24′ E) and the University of Tennessee’s Organic Crops Unit near Knoxville (TN, USA; 35°53′ N, 83°56′ W), respectively. Prior to use, both soil samples are air-dried and sieved to pass 2-mm mesh. According to the international soil texture classification, the brown earth is a silty clay loam (18% sand, 54% silt, and 28% clay) with pH 6.3, organic matter content 17.5 g kg^−1^, and citrate-bicarbonate-dithionite extractable iron 11 g kg^−1^. The red earth is a clay loam (30% sand, 41% silt, 29% clay) with pH 6.9, organic matter content 18.8 g kg^−1^, and citrate-bicarbonate-dithionite extractable iron 55 g kg^−1^. 

Water-dispersible colloids (<10 μm) used in this study were extracted by centrifugation from the brown earth using the method described in Bao et al. [35]. Specifically, sampled soil was air-dried and passed through a 100-mesh stainless steel sieve. Then, 20 g of the sieved soil samples were placed in a centrifuge tube, followed by an addition of 600 mL of deionized water. The mixed slurry was shaken for 1 h and further centrifuged at 54 g for 3.5 min (Universal 320, Hettich, Germany). The above procedure was repeated twice for each 20 g of the soil samples. Finally, the colloid particles <10 μm remaining in the suspension were decanted, freeze-dried, and stored in glass vials at room temperature (21 ± 2 °C) before use. CNCs in water suspension was purchased from the University of Maine (Orono, ME, USA) and had a solid content of 65 g kg^−1^ and a crystallinity index of 81%. Their dimensions were 200–400 nm in length and smaller than 10 nm in width.

### 2.2. Column Experiments

The experimental setup consisted of a stainless steel column (1.1-cm in inner diameter, 10.5-cm in height), a peristaltic pump (BT100-2J, Baoding Longer Precision Pump Co., Ltd., Baoding, Hebei, China), and a fraction collector (CF-2, Spectrum Laboratories Inc., Los Angeles, CA, USA). The chemicals employed in this study included NaBr, NaCl, HCl, and NaOH, which were purchased from Sinopharm Chemical Reagent Co., Ltd. (Shanghai, China). NaBr was used as a conservative tracer, NaCl served as background solution, and HCl and NaOH were used to adjust the pH of the input solution. A 0.5-cm quartz sand layer and a 0.5-mm glass fiber filter were placed at the top and bottom of the column, respectively, to prevent possible blockage of the tubing by porous media grains during the transport experiments. Thirteen one-dimensional vertical column experiments were performed under steady-state saturated flow conditions. Among them, four experiments were conducted to investigate the mobility of CNCs in oxide-free sand and hematite-coated sand at different ionic strengths (5 mM and 50 mM). Four experiments focused on examining CNCs transport in natural soils (red earth and brown earth) at different ionic strengths (5 mM and 50 mM). Four experiments were designed to investigate the effect of soil colloids on CNCs transport through hematite-coated sand and brown earth at the above two ionic strengths. One experiment was conducted to examine the mobility of soil colloids in brown earth at higher ionic strength (50 mM). Detailed column experimental conditions are provided in Table 1. 

The columns were dry-packed with the sand or the soils at 1-cm increment, and tapped down after each addition to tighten the medium. After packing, CO_2_ was pumped into the columns for 4 h to replace entrapped air in soil pores. The columns were then saturated and flushed by injecting ~20 pore volumes of a deaerated background solution (5 mM or 50 mM NaCl at pH 7.0) upward through the peristaltic pump. After the effluent stabilized in terms of pH and flow rate, 20 pore volumes of experimental solution, which contained a conservative tracer (30 mg L^−1^ NaBr) and a stable CNCs suspension (200 mg L^−1^, with or without soil colloid at 50 mg L^−1^), was introduced into the column before the column was eluted with the background solution of a given ionic strength (5 mM or 50 mM). The effluent solution was collected using the fraction collector at regular time intervals throughout the experiments. The injection of the tracer served for estimating the dispersion coefficients (*D*) by fitting the standard convection-dispersion equation to the bromide breakthrough curves. The bromide concentrations in the effluent were determined using ion chromatography (ICS 5000, Dionex, Sunnyvale, CA, USA).

### 2.3. Analysis of CNCs and Soil Colloids

CNCs concentrations in the effluent were determined using a TOC analyzer (multi N/C 3000, Analytik Jena AG, Germany). The diameters and zeta potentials of CNCs were measured using a Zetasizer (Nano-ZS, Malvern Instruments Ltd., Malvern, UK) in the background solutions used for the column experiments. The concentration of water-dispersible soil colloids was determined based on a calibration curve established for soil colloids concentration on the absorbance at 450 nm (Victor X, PerkinElmer, Waltham, MA, USA). The calibration curve was established by diluting the 100 mg L^−1^ of dispersive soil colloids suspension, which was linear within the range of 0–50 mg L^−1^.

The concentrations of dissolved organic carbon (DOC) and colloids were monitored to detect their potential release from the packed soils during the pre-flush with background solution (i.e., free of CNCs and colloids). The injection of experimental solution, which contained CNCs and/or soil colloids, started when the effluent concentrations of DOC and soil colloids leveled off after ~13 pore volumes. The breakthrough concentrations of injected CNCs and soil colloids were obtained after deduction of the concentrations of released native colloids. Preliminary experimental results showed that CNCs (200 mg L^−1^) had a minimal absorbance (<0.003) at 450 nm, and thus the influence of CNCs on the measurement of soil colloid concentrations at 450 nm was negligible.

### 2.4. Theoretical Consideration

#### 2.4.1. Transport Modeling

The transport behavior of CNCs through porous media under steady-state saturated flow conditions can be described by a convection-dispersion equation (CDE) including a term for first-order particle deposition (Kretzschmar et al. [48]):

(1) $$\frac{\partial C}{\partial t}=\;D\;\frac{\partial^{2}C}{\partial x^{2}}\;-\;v\frac{\partial C}{\partial x}-\;\mathrm{kC}$$

where C (mg L^−1^) is CNCs concentration in the liquid phase, D (cm^2^ h^−1^) is the hydrodynamic dispersion coefficient, v (cm h^−1^) is the Darcy velocity, and k is the CNCs deposition rate coefficient. The value of kC is estimated by:

(2) $$\mathrm{kC}=\;\frac{\rho_{b}}{\theta }\frac{\partial S}{\partial t}{\;=\;\Psi k}_{\mathrm{dep}}C\;-\;\frac{\rho_{b}}{\theta }k_{\det}S$$

where ρ_b_ (g cm^−3^) is the bulk density of the porous medium, S (μg g^−1^) is CNCs concentration on porous media, k_dep_ (h^−1^) is the deposition coefficient, k_det_ (h^−1^) is the first-order detachment rate coefficient, and Ψ is a dimensionless deposition function for CNCs, which accounts for time- and depth-dependent deposition behaviors, as calculated using the following formula [49,50]:

(3) $$\Psi \;=\;(1\;-\;\frac{S}{S_{\max}}){\left(\frac{d_{c}\;+\;x}{d_{c}}\right)}^{-\beta }$$

where d_c_ (cm) is the median particle diameter of the porous medium, x (cm) is the down gradient distance from the porous medium inlet, S_max_ (μg g^−1^) is the maximum concentration of deposited CNCs, and β is an empirical factor, with an optimal value of 0.432 as suggested by Bradford et al. (2003) [49]. The above model was applied to simulate the breakthrough curves by simultaneously inverse fitting of D, S_max_, k_dep_, and k_det_ using the Levenberg-Marquardt nonlinear least squares optimization algorithm in HYDRUS-1D. The uniqueness of the fitted results was verified by re-running the program with different initial parameter estimates.

#### 2.4.2. Calculation of the Maximum Travel Distance

Based on the above assumption of the first-order deposition kinetics, CNCs breakthrough curves can be evaluated by calculating a clean-bed filter coefficient described as [51]:

(4) $$\lambda_{0}\;=\;-\frac{1}{L}\;\ln\left(\frac{C_{f}}{C_{0}}\right)$$

where L is the column length, C_0_ is the initial effluent concentration of CNCs, C_f_ is the effluent concentration of CNCs after the breakthrough curve has reached a plateau. The deposition rate coefficient *k* can be estimated as below:

(5) $$k\;=\;\lambda_{0}v_{p}\;=\;\lambda_{0}\;\frac{L}{t_{p}}\;=\;-\frac{1}{t_{p}}\;\ln\left(\frac{C_{f}}{C_{0}}\right)$$

where $t_{p}$ represents the average travel time of CNCs through the column, the term $C_{f}/C_{0}$ equals the fraction of CNCs recovered at the column outlet after the breakthrough curve has reached a plateau. Thus the maximum travel distance of the CNCs, defined by the distance at which 99% of them have been removed from the solution, can be estimated by:

(6) $$L_{\max}\;=\;-\frac{v_{p}}{k}\;\ln\left(\frac{C_{f}}{C_{0}}\right)$$

where $C_{f}/C_{0}$ was set at 0.001.

#### 2.4.3. DLVO Interaction Energy Calculations

To interpret the mechanisms controlling attachment of CNCs and soil colloids on sand or soil surfaces, the DLVO interaction energies of a CNC or a soil colloid with a sand collector surface were calculated at ionic strengths of 5 mM and 50 mM. The total DLVO interaction energy (*U*) is the sum of van der Waals (VDW) attraction, double layer (DL) energy, and short-range repulsion [52,53]. The short-range repulsion was considered by determining Born (BR) potential energy [54]. We assumed the CNCs or soil colloids as spheres and sand surfaces to be planar. The surface element integration technique (SEI) developed by Bhattacharjee and Elimelech was used to calculate the DLVO energies for the sphere-planar surface interaction configurations [55]. Briefly, the CNC nanoparticle or soil colloid and collector surface were discretized into small area elements. The total interaction energy was obtained by summing the differential interaction energy between each pair of area elements on the colloid and the surface. The expressions developed by Hamaker, Hogg et al. and Oliveira were used to calculate VDW, DL, and BR differential energies, respectively [56,57,58].

The interaction energy between a CNC or soil colloid and the iron oxide surface was also determined to evaluate the influence of surface charge heterogeneity on attachment. We only calculated the DLVO energies between a CNC or soil colloid and the surface completely consisting of iron oxide because the coated hematite sizes were much larger than the CNC diameters. It should be noted that although the CNCs aggregated at 50 mM, we used the average hydrodynamic diameter (i.e., ~276.53 nm) measured at 5 mM for the interaction energy calculations since Lin and Wiesner demonstrated that the interaction energy for the aggregated colloids was on the same order of magnitude as those for the primary particles of the aggregates and was significantly smaller than that for an equivalent sphere (e.g., defined by the gyration radius of the aggregate) [59]. The calculated DLVO energies here did not address the potential impact of aspect ratio. The relatively high aspect ratios did not affect the trend of the energy barrier for solid (200–1000) and hollow (25–1000) cylinders, whereas a relatively low aspect ratio produces a non-monotonic trend of energy barrier for solid (2–100) and hollow (2–10) cylinders [60].

## 3. Results and Discussion

### 3.1. Transport of Bromide

**Figure 2:**
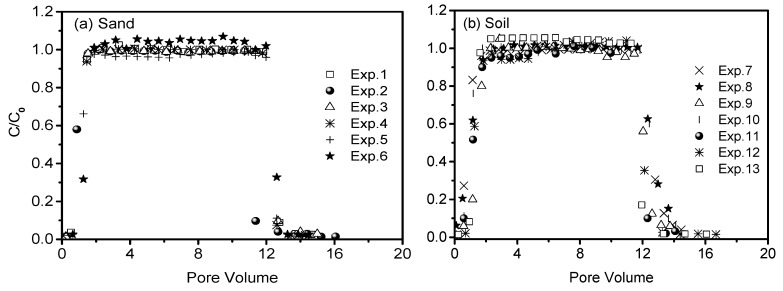
Breakthrough curves of bromide ion from columns repacked with sand (**a**) and soil (**b**) under saturated flow conditions.

Breakthrough curves of conservative bromide from all column experiments were illustrated in Figure 2. The reproducibility indicated that the column systems were stable and similar in the hydrodynamic conditions among different column experiments. The breakthrough curves of bromide were fitted with classic convection-dispersion equation to obtain the dispersion coefficients (D), which were subsequently used to simulate the transport behavior of CNCs. Discrepancies in the values of D are attributed to the slight differences in the flow velocity and/or the bulk density of packed porous media (Table 1). 

### 3.2. Transport of CNCs in Hematite-Coated Sand

**Figure 3:**
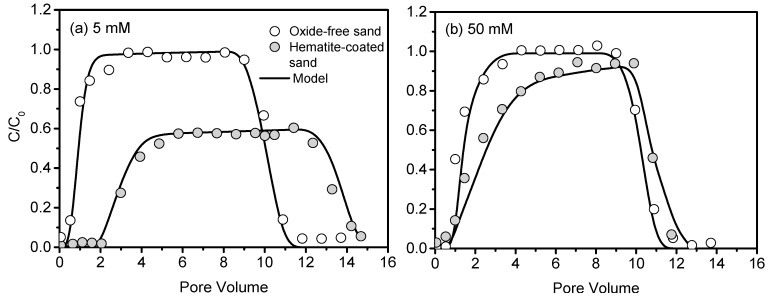
Effect of iron oxide (hematite) on the transport of CNCs through sand at 5 mM (**a**) and 50 mM (**b**).

The retention of CNCs was minimal during the transport through oxide-free sand, with the maximum relative effluent concentration (max *C/C*_0_) larger than 0.98 and the effluent percentages at least 99% at both ionic strengths (5 mM and 50 mM). Hematite coating hindered breakthrough of CNCs compared with oxide-free sand by ~2.5 pore volumes and ~0.1 pore volume, decreased max *C/C*_0_ by ~0.4 and ~0.1, and decreased effluent percentages by ~48% and ~14% at 5 mM and 50 mM, respectively (Figure 3, Table 2). The discrepancies were attributed to the change in sand surface properties after the hematite coating. For instance, the hematite coating increased the specific surface area of sand from 0.15 m^2^ g^−1^ to 0.35 m^2^ g^−1^ and increased the zeta potential of sand from −47 mV to 4 mV. As a result, the increased electrostatic attraction between hematite-coated sand and oppositely charged CNCs hindered the transport of CNCs. Different from the symmetric breakthrough curves predicted from colloid filtration theory, the observed breakthrough and elution curves of CNCs tended to be asymmetric in the hematite-coated sand as ionic strength increased. This trend is commonly regarded as an indicator of blocking effect, which represents the progressive occupation of available deposition sites on the collector surfaces over time due to dynamic blocking [61].

The one-dimensional CDE model provided a good description for CNCs breakthrough (Table 1). Consistent with the breakthrough results, the maximum solid deposition concentration (S_max_) and deposition coefficient (k_dep_) of CNCs in oxide-free sand were low (less than 0.2), indicating that the oxide-free sand had small retention capacity for CNCs at both ionic strengths. In comparison, the values of S_max_ and k_dep_ of CNCs in hematite-coated sand increased ~72 fold and ~7 fold at 5 mM, and increased ~6 fold and ~2 fold at 50 mM, respectively. The smaller values of S_max_ and k_dep_ at higher ionic strength (50 mM) indicated that the retention capacity of hematite-coated sand decreased, which was favorable for CNCs transport. Another index of CNCs mobility in sands is the maximum transport distance L_max_ (Table 2). Consistent with the small S_max_ and k_dep_, the largest value of L_max_ of CNCs in the oxide-free sand column were 7245 cm at 5 mM and 5573 cm at 50 mM. In contrast, these values in the hematite-coated sand decreased to 129 cm and 622 cm at 5 mM and 50 mM, respectively.

**Figure 4:**
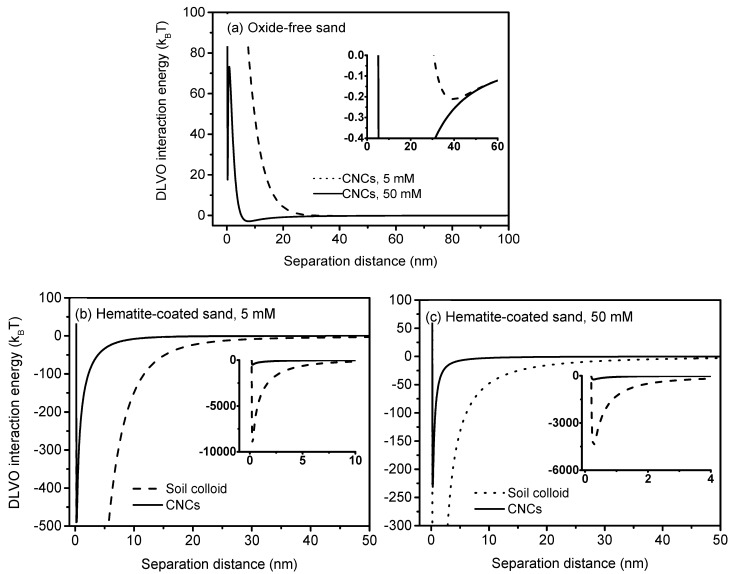
Predicted DLVO interaction energy profiles between CNCs and oxide-free sand (**a**) and between CNCs/soil colloids and hematite-coated sand at 5 mM (**b**) and 50 mM (**c**). Subgraphs are detailed view of primary minima or secondary minima.

The above large discrepancies of CNCs breakthrough behavior can be attributed to changes in sand surface characteristics after the coating [62]. Hematite coating turned the DL interaction from repulsion to attraction between CNCs and the sand [63], resulting in favorable retention of CNCs. The nonexistence of the primary energy minimum and the shallow secondary energy minimum (−0.21 *k_B_T* at 5 mM and −2.87 *k_B_T* at 50 mM) indicated the minimal attachment of CNCs on the oxide-free sand (*k_B_* is Boltzmann constant, *T* is absolute temperature of fluid), consistent with the breakthrough results (Figure 3 and Figure 4a). In contrast, there was no primary energy barriers and secondary energy minimum on the hematite-coated sand, suggesting that attractive forces dominated the interaction between CNCs and sand. The presence of deep primary energy minimum (−482.35 k_B_T at 5 mM and −228.73 k_B_T at 50 mM) indicated that irreversible retention of CNCs on hematite-coated sand was likely under the conditions of this study (Figure 4b,c). It should be noted that CNCs particles are solid cylinders with a relatively low aspect ratio (<100), which produces a non-monotonic decrease trend of energy barrier, favorable for CNCs retention. In this study, the potential impact of aspect ratio might be small due to the nonexistence of both the primary energy minimum on oxide-free sand and energy barrier on hematite-coated sand. 

Increasing ionic strength exerted different effects on CNCs transport through clean sand and hematite-coated sand. In the oxide-free sand, CNCs exhibited high mobility with almost complete breakthrough at both ionic strengths (5 mM and 50 mM), indicating minimal effect of electrostatic interactions. This is due to existence of significant energy barriers and shallow secondary minima at those ionic strengths (Figure 4a). Hence, the attachment in both primary and secondary minimum was inhibited. In comparison, the effluent percentages of CNCs from the hematite-coated sand columns increased from ~52% to ~86% in response to the increase in ionic strength from 5 to 50 mM, demonstrating that higher ionic strength was favorable to the transport of CNCs through the hematite-coated sand. In order to investigate the relevant mechanism, we measured the zeta potentials of CNCs and sands at different ionic strengths. At 5 mM, the zeta potentials of CNCs, oxide-free sand, and hematite-coated sand were −39 mV, −47 mV, and 4 mV, respectively (Table 3). Obviously, the strong electrostatic repulsion between CNCs and like-charged oxide-free sand favored CNCs transport, whereas the electrostatic attraction between CNCs and opposite-charged hematite-coated sand hindered the transport of CNCs. When ionic strength increased from 5 mM to 50 mM, the absolute zeta potentials of CNCs, oxide-free sand, and hematite-coated sand decreased by 11 mV, 9 mV and 3 mV, respectively. Thus, CNCs experienced a decrease in electrostatic repulsion from oxide-free sand (increasing attachment) and decrease in electrostatic attraction from hematite-coated sand (decreasing attachment). It is worth noting that the mobility of CNCs in oxide-free sand and in hematite-coated sand was still high, despite the ~153 nm increase in the diameter of CNCs as the ionic strength increased from 5 mM to 50 mM (Table 3). This phenomenon indicated a negligible effect of mechanical straining (i.e., blocking by pores) because the diameter ratio of CNCs to sand (~0.0006) was far below the occurrence value for mechanical straining (0.002). Thus, the aggregation of CNCs had minimal effect on their transport through the sands.

### 3.3. Transport of CNCs in Soils

**Figure 5:**
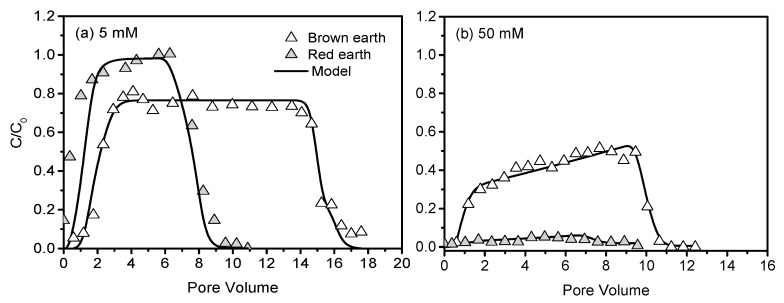
Breakthrough curves of CNCs from natural loamy soils at 5 mM (**a**) and 50 mM (**b**).

CNCs demonstrated higher mobility in the natural soils. At 5 mM, the max *C/C*_0_ was ~1.0 in red earth and ~0.8 in brown earth, and the corresponding effluent percentages were 96% and 80%, respectively (Figure 5a, Table 2). In comparison, red earth was much more sensitive to the change in ionic strength than brown earth. When the ionic strength increased from 5 to 50 mM, the max *C/C*_0_ of CNCs decreased from ~1.0 to ~0.1 in red earth and from ~0.8 to ~0.5 in brown earth (Figure 5b). As a result, the effluent percentages decreased by ~92% in red earth and ~42% in brown earth. Meanwhile, the value of L_max_ decreased by ~10 fold in red earth and ~3 fold in brown earth (Table 2). Such a large effect of ionic strength on the transport of CNCs in red earth than in brown earth was attributed to the differences in soil properties and soil surface heterogeneity. Compared to brown earth, red earth had finer texture and higher iron oxide content, suggesting higher surface heterogeneity. Shen et al. (2013) found that a critical size of positively charged patch was necessary for colloid attachment at a given ionic strength, and the needed critical size decreased with increasing ionic strength, which was favorable for the attachment [64]. This implies that large surface heterogeneities caused by metal oxides and organic matter are favorable for CNCs attachment at higher solution ionic strength. Unfortunately, we cannot evaluate this possibility because we do not have the heterogeneity data of the experimental soils. 

In addition, coagulation of CNCs (e.g., the average diameter of CNCs increased by 153 nm) might contribute to the ionic strength effect since larger colloids are more sensitive to the surface heterogeneity [53,64,65]. In general, we speculate that the higher surface heterogeneity of red earth grains is responsible for the larger ionic strength effect compared to brown earth.

### 3.4. Effect of Soil Colloids on CNCs Transport

**Figure 6:**
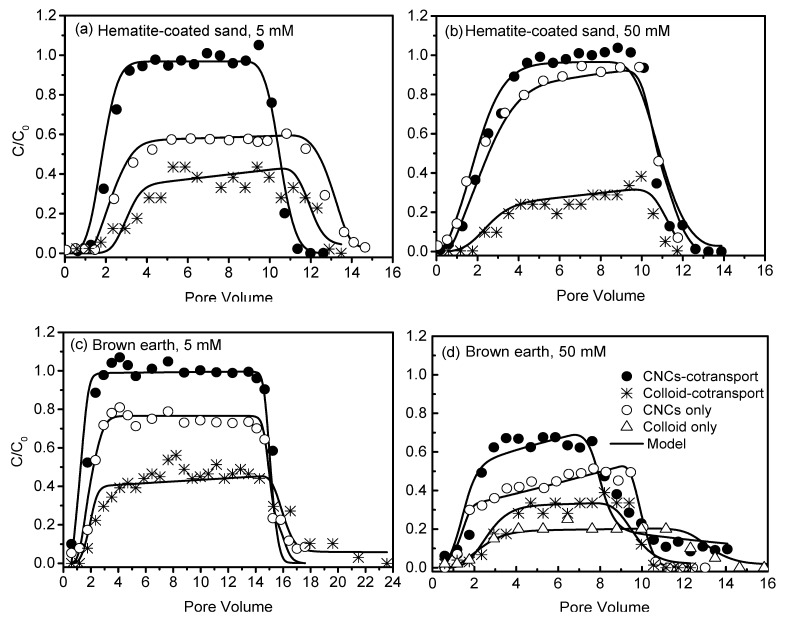
Effect of soil colloids (50 mg L^−1^) on the transport of CNCs (200 mg L^−1^) through hematite-coated sand at 5 mM (**a**) and 50 mM (**b**) and brown earth at 5 mM (**c**) and 50 mM (**d**).

Results from the co-transport experiments of CNCs and soil colloids in hematite-coated sand indicated that existence of soil colloids at 50 mg L^−1^ shortened the initial breakthrough time of CNCs thereby promoting their transport (Figure 6a,b). The effluent percentage of CNCs increased by ~42% at 5 mM and ~6% at 50 mM (Table 2). Under the same experimental condition, the effluent percentages of soil colloids were ~38% at 5 mM and ~19% at 50 mM. Similarly, the presence of soil colloids increased the effluent percentage of CNCs from the brown earth by ~18% at 5 mM and ~31% at 50 mM (Figure 6c,d). Meanwhile, the effluent percentages of soil colloids were ~48% at 5 mM and ~31% at 50 mM (Table 2). The larger facilitated effect on the transport of CNCs at 50 mM than 5 mM might be a result of reduction of dispersive pathways and/or increase in preferential pathways for CNCs after a portion of narrow pore throats in the soil were blocked by aggregated colloids under elevated ionic strength condition. On the other hand, the presence of CNCs at 200 mg L^−1^ also promoted the transport of soil colloids through brown earth with the effluent percentage of soil colloids increased by ~12% at 50 mM (Figure 6d). These results demonstrate that the facilitated effect of soil colloids on CNCs transport was more pronounced than the influence of CNCs on the transport of soil colloids.

Results from the co-transport experiments in soil demonstrated that nearly all CNCs particles broke through the soil at 5 mM with the effluent percentage reaching 99%, while approximately half of the soil colloids were retained with the effluent percentage only 48%. This result suggests the non-existence of mutual attachment between CNCs and soil colloids at low ionic strength. The reason for the facilitated effect of soil colloids on CNCs transport was not due to their carrier role, but because soil colloids acted as obstacles for the attachment of CNCs to the surface of soils. In this study, both soil colloids and CNCs were negatively charged with similar zeta potentials (Table 3). The soil surface was electronegative as a whole with existence of positively charged patches of iron oxides, which were five times as great as on red earth than on brown earth. Once the soil colloids and CNCs particles attached to and occupied the positively charged sites, the electronegativity of soil surface increased. As a result, the electrostatic repulsion between soil colloids/CNCs and soil surface increased to facilitate the transport of both particles. The breakthrough of soil colloids alone was low, probably due to the mechanical straining, as the average diameter ratio of soil colloids to soil particles (0.005) exceeds the threshold value of straining (0.002). Above results were consistent with numerical modeling results, suggesting that the presence of soil colloids could hinder the retention of CNCs on favorable soil sites (Table 1). 

## 4. Conclusions

This study investigated the effects of ionic strength, iron oxides (hematite), and soil colloids on the transport behavior of CNCs in a model sand and two natural soils. Results indicated that CNCs had high mobility in oxide-free quartz sand due mainly to the strong electrostatic repulsion between negatively charged CNCs and same charged sand. The existence of hematite hindered the transport of CNCs, especially under low ionic strength, mainly due to the strong electrostatic attraction between CNCs and oppositely charged hematite. Results from the DLVO interactions indicated that CNCs suffered strong repulsive forces on oxide-free sand and experienced strong attractive forces on hematite-coated sand. CNCs showed a relatively high breakthrough from natural loam soils with effluent percentage higher than 80% and maximum travel distance farther than 70 m under low ionic strength (5 mM). The mobility of CNCs was much more dependent on solution ionic strength in natural soils than in sand. The increase in ionic strength and the presence of iron oxides on soil surfaces hindered CNCs transport. Mass recovery results indicated that CNCs and soil colloids promoted their transport mutually, with the facilitated effect of soil colloids on CNCs transport more than 2 fold larger than the promotion of CNCs on the transport of soil colloids. The results of this study suggest that CNCs has great potential for being utilized to elute contaminants from contaminated soils due to their high dispersivity and mobility in natural soils. Future investigation should evaluate the contaminant adsorption efficiency of CNCs and their mobility under unsaturated flow conditions.

## Figures and Tables

**Table 1:** Experimental conditions and fitted parameters for column experiments.

Exp. #	Porous Media	CNCs (mg L^−1^)	Colloid (mg L^−1^)	I_S_ (mM)	D (cm^2^ h^−1^)	ρ_b_ (g cm^−3^)	θ (%)	V_p_ (cm h^−1^)	S_max_ (mg L^−1^)	k_dep_ (h^−1^)	*k*_det_ (h^−1^)	R^2^
1	Oxide-free sand	200	0	5	0.14	1.72	36.24	14.80	0.05	0.14	4 × 10^−4^	0.98
2		200	0	50	0.15	1.72	36.21	14.75	0.07	0.15	2 × 10^−3^	0.95
3	Hematite-coated sand	200	0	5	0.36	1.72	36.40	14.75	3.61	0.92	5 × 10^−5^	0.97
4		200	0	50	0.24	1.72	36.43	14.75	0.40	0.31	5 × 10^−5^	0.91
5		200	50	5	0.13	1.49	43.82	11.40	0.12	0.03	5 × 10^−6^	0.98
6		200	50	50	0.23	1.49	43.84	13.81	0.09	0.03	5 × 10^−3^	0.97
7	Red earth	200	0	5	0.23	1.15	57.40	9.32	0.17	0.03	4 × 10^−3^	0.87
8		200	0	50	0.51	1.15	57.42	12.19	15.94	5.55	4 × 10^−2^	0.63
9	Brown earth	200	0	5	0.15	1.37	48.24	11.44	0.08	0.05	2 × 10^−3^	0.98
10		200	0	50	0.44	1.33	46.61	9.34	3.56	2.87	4 × 10^−3^	0.85
11		200	50	5	0.15	1.33	47.03	12.02	0.04	0.17	2 × 10^−3^	0.89
12		200	50	50	0.50	1.33	47.01	12.06	1.57	1.10	3 × 10^−1^	0.89
13		0	50	50	0.32	1.33	46.64	11.44	-	-	-	-

I_s_: ionic strength; D: dispersion coefficient; ρ_b_: bulk density; θ: porosity; V_p_: pore velocity; S_max_: maximum concentration of deposited particles; k_dep_: first-order deposition coefficient; k_det_: first-order detachment coefficient; R^2^: coefficient of determination.

**Table 2:** Mass recoveries of CNCs and soil colloids during the transport experiments.

Exp.#	Porous Media	CNCs (mg L^−1^)	Soil Colloids (mg L^−1^)	I_s_ (mM)	CNCs				Soil Colloids	
					M_eff_ (%)	M_ret_ (%)	M_tot_ (%)	L_max_ (cm)	M_eff_ (%)	L_max_ (cm)
1	Oxide-free sand	200	0	5	99.78	0.68	100.46	7245	-	-
2		200	0	50	99.56	1.64	101.20	5573	-	-
3	Hematite-coated sand	200	0	5	51.58	42.80	94.38	129	-	-
4		200	0	50	85.56	13.19	98.75	622	-	-
5		200	50	5	93.67	7.87	101.54	3623	38.37	79.09
6		200	50	50	91.44	10.40	101.84	7245	19.47	60.38
7	Red earth	200	0	5	96.31	1.67	97.98	2415	-	-
8		200	0	50	4.37	94.17	98.54	24	-	-
9	Brown earth	200	0	5	80.39	18.20	98.59	325	-	-
10		200	0	50	37.94	52.54	90.48	102	-	-
11		200	50	5	98.79	3.14	101.93	7245	47.94	98.71
12		200	50	50	69.19	15.20	84.39	181	30.76	65.27
13		0	50	50	-	-	-	-	19.02	45.00

I_s_: ionic strength; M_eff_: effluent percentage; M_ret_: retention percentage; M_tot_: total percentage; L_max_: the maximum travel distance.

**Table 3:** Measured sizes and/or zeta potentials of colloids and porous media.

	I_s_ (mM)	d_h_ (nm)	ε (mV)
CNCs	5	276.53 ± 22.82	−39.26 ± 9.04
	50	429.33 ± 76.11	−28.03 ± 2.28
Oxide−free sand	5	−	−47.20 ± 3.75
	50	−	−37.91 ± 4.72
Hematite−coated sand	5	−	3.87 ± 1.04
	50	−	0.47 ± 0.10
Soil colloids	5	−	−40.07 ± 5.21
	50	−	−30.86 ± 1.28

I_s_: ionic strength; d_h_*:* Hydrodynamic diameter; ε: zeta potential.

## References

[1] Jiang D., Zeng G., Huang D., Chen M., Zhang C., Huang C., Jia W. (2018). Remediation of contaminated soils by enhanced nanoscale zero valent iron. Environ. Res..

[2] Li Q., Chen X.J., Chen X., Jin Y., Zhuang J. (2019). Cadmium removal from soil by fulvic acid-aided hydroxyapatite nanofluid. Chemosphere.

[3] Chen M., Tao X.Y., Wang D.J., Xu Z.B., Xu X.Y., Hu X.F., Xu N., Cao X.D. (2019). Facilitated transport of cadmium by biochar-Fe_3_O_4_ nanocomposites in water-saturated natural soils. Sci. Total Environ..

[4] Wang D.J., Bradford S.A., Harvey R.W., Gao B., Cang L., Zhou D.M. (2012). Humic acid facilitates the transport of ARS-labeled hydroxyapatite nanoparticles in iron oxyhydroxide-coated sand. Environ. Sci. Technol..

[5] Zhang W., Rattanaudompol U.S., Li H., Bouchard D. (2013). Effects of humic and fulvic acids on aggregation of aqu/nC (60) nanoparticles. Water Res..

[6] Zhang Z., Li M., Chen W., Zhu S., Liu N., Zhu L. (2010). Immobilization of Lead and Cadmium from aqueous solution and contaminated sediment using nano-hydroxyapatite. Environ. Pollut..

[7] Bystrzejewska-Piotrowska G., Golimowski J., Urban P.L. (2009). Nanoparticles: Their potential toxicity, waste and environmental management. Waste Manag..

[8] Kahru A., Dubourguier H.-C., Blinova I., Ivask A., Kasemets K. (2008). Biotests and Biosensors for Ecotoxicology of Metal Oxide Nanoparticles: A Minireview. Sensors.

[9] Besseling E., Wang B., Lürling M., Koelmans A.A. (2014). Nanoplastic Affects Growth of *S. obliquus* and Reproduction of *D. magna*. Environ. Sci. Technol..

[10] Boxall A.B.A., Karen T., Qasim C. (2007). Engineered nanomaterials in soils and water: How do they behave and could they pose a risk to human health?. Nanomedicine.

[11] Cowie H., Magdolenova Z., Saunders M., Drlickova M., Carreira S.C., Kenzaoi B.H., Gombau L., Guadagnini R., Lorenzo Y., Walker L. (2015). Suitability of human and mammalian cells of different origin for the assessment of genotoxicity of metal and polymeric engineered nanoparticles. Nanotoxicology.

[12] Fadri G., Bernd N. (2011). The release of engineered nanomaterials to the environment. J. Environ. Monit..

[13] Griffitt R.J., Luo J., Gao J., Bonzongo J.C., Barber D.S. (2008). Effects of particle composition and species on toxicity of metallic nanomaterials in aquatic organisms. Environ. Toxicol. Chem..

[14] Hanna S., Miller R., Lenihan H. (2014). Accumulation and Toxicity of Copper Oxide Engineered Nanoparticles in a Marine Mussel. Nanomaterials.

[15] Lehner R., Weder C., Petri-Fink A., Rothen-Rutishauser B. (2019). Emergence of nanoplastic in the environment and possible impact on human health. Environ. Sci. Technol..

[16] Grishkewich N., Mohammed N., Tang J., Tam K.C. (2017). Recent advances in the application of cellulose nanocrystals. Curr. Opin. Colloid Interface Sci..

[17] Lam E., Male K.B., Chong J.H., Leung A.C.W., Luong J.H.T. (2012). Applications of functionalized and nanoparticle-modified nanocrystalline cellulose. Trends Biotechnol..

[18] Peng B.L., Dhar N., Liu H.L., Tam K.C. (2011). Chemistry and applications of nanocrystalline cellulose and its derivatives: A nanotechnology perspective. Can. J. Chem. Eng..

[19] Tingaut P., Zimmermann T., Sèbe G. (2012). Cellulose nanocrystals and microfibrillated cellulose as building blocks for the design of hierarchical functional materials. J. Mater. Chem..

[20] Du L.Y., Arnholt K., Ripp S., Sayler G., Wang S.Q., Liang C.H., Wang J.K., Zhuang J. (2015). Biological toxicity of cellulose nanocrystals (CNCs) against the luxCDABE-based bioluminescent bioreporter Escherichia coli 652T7. Ecotoxicology.

[21] Lu Q.L., Cai Z.H., Lin F.C., Tang L.R., Wang S.Q., Huang B. (2016). Extraction of cellulose nanocrystals with a high yield of 88% by simultaneous mechanochemical activation and phosphotungstic acid hydrolysis. ACS Sustain. Chem. Eng..

[22] Azizi Samir M.A.S., Alloin F., Dufresne A. (2005). Review of recent research into cellulosic whiskers, their properties and their application in nanocomposite field. Biomacromolecules.

[23] Carpenter A.W., de Lannoy C.-F., Wiesner M.R. (2015). Cellulose Nanomaterials in Water Treatment Technologies. Environ. Sci. Technol..

[24] Meng Y.J., Wu Q., Young T.M., Huang B., Wang S.Q., Li Y.J. (2017). Analyzing three-dimensional structure and geometrical shape of individual cellulose nanocrystal from switchgrass. Polym. Compos..

[25] Wu Q., Li X.W., Li Q., Wang S.Q., Luo Y. (2019). Estimation of aspect ratio of cellulose nanocrystals by viscosity measurement: Influence of aspect ratio distribution and ionic strength. Polymers.

[26] Anirudhan T.S., Deepa J.R., Christa J. (2016). Nanocellulose/nanobentonite composite anchored with multi-carboxyl functional groups as an adsorbent for the effective removal of Cobalt(II) from nuclear industry wastewater samples. J. Colloid Interface Sci..

[27] Hasani M., Cranston E.D., Westman G., Gray D.G. (2008). Cationic surface functionalization of cellulose nanocrystals. Soft Matter.

[28] Kloser E., Gray D.G. (2010). Surface Grafting of Cellulose Nanocrystals with Poly (ethylene oxide) in Aqueous Media. Langmuir.

[29] Zhou C., Lee S., Dooley K., Wu Q. (2013). A facile approach to fabricate porous nanocomposite gels based on partially hydrolyzed polyacrylamide and cellulose nanocrystals for adsorbing methylene blue at low concentrations. J. Hazard. Mater..

[30] Zhou C., Wu Q., Lei T., Negulescu I.I. (2014). Adsorption kinetic and equilibrium studies for methylene blue dye by partially hydrolyzed polyacrylamide/cellulose nanocrystal nanocomposite hydrogels. Chem. Eng. J..

[31] Liu P., Sehaqui H., Tingaut P., Wichser A., Oksman K., Mathew A.P. (2014). Cellulose and chitin nanomaterials for capturing silver ions (Ag^+^) from water via surface adsorption. Cellulose.

[32] Liu P., Borrell P.F., Božič M., Kokol V., Oksman K., Mathew A.P. (2015). Nanocelluloses and their phosphorylated derivatives for selective adsorption of Ag^+^, Cu^2+^ and Fe^3+^ from industrial effluents. J. Hazard. Mater..

[33] Mahfoudhi N., Boufi S. (2017). Nanocellulose as a novel nanostructured adsorbent for environmental remediation: A review. Cellulose.

[34] Sheikhi A., Safari S., Yang H., Tg V.D.V. (2015). Copper removal using electrosterically stabilized nanocrystalline cellulose. ACS Appl. Mater. Int..

[35] Bao Q., Lin Q., Tian G., Wang G., Yu J., Peng G. (2011). Copper distribution in water-dispersible colloids of swine manure and its transport through quartz sand. J. Hazard. Mater..

[36] Zhong L., Fu S., Peng X., Zhan H., Sun R. (2012). Colloidal stability of negatively charged cellulose nanocrystalline in aqueous systems. Carbohyd. Polym..

[37] Singh K., Arora J., Jai Mangal Sinha T., Srivastava S. (2014). Functionalization of nanocrystalline cellulose for decontamination of Cr(III) and Cr(VI) from aqueous system: Computational modeling approach. Clean Technol. Environ. Policy.

[38] Liang Y., Zhou J., Dong Y., Klumpp E., Šimůnek J., Bradford S.A. (2019). Evidence for the critical role of nanoscale surface roughness on the retention and release of silver nanoparticles in porous media. Environ. Pollut..

[39] Shen C.Y., Jin Y., Zhuang J., Li T.T., Xing B.S. (2020). Role and importance of surface heterogeneities in transport of particles in saturated porous media. Crit. Rev. Environ. Sci. Technol..

[40] Torkzaban S., Bradford S.A., van Genuchten M.T., Walker S.L. (2008). Colloid transport in unsaturated porous media: The role of water content and ionic strength on particle straining. J. Contam. Hydrol..

[41] Wang D.J., Bradford S.A., Harvey R.W., Hao X.Z., Zhou D.M. (2012). Transport of ARS-labeled hydroxyapatite nanoparticles in saturated granular media is influenced by surface charge variability even in the presence of humic acid. J. Hazard. Mater..

[42] Lin S., Cheng Y., Bobcombe Y., Jones K.L., Liu J., Wiesner M.R. (2011). Deposition of silver nanoparticles in geochemically heterogeneous porous media: Predict-ing affinity from surface composition analysis. Environ. Sci. Technol..

[43] Wang L., Li J., Jiang Q., Zhao L. (2012). Water-soluble Fe_3_O_4_ nanoparticles with high solubility for removal of heavy-metal ions from waste water. Dalton Trans..

[44] Yan J., Lazouskaya V., Jin Y. (2016). Soil colloid release affected by dissolved organic matter and redox conditions. Vadose Zone J..

[45] Zhu Y., Ma L.Q., Dong X., Harris W.G., Bonzongo J., Han F. (2014). Ionic strength reduction and flow interruption enhanced colloid-facilitated Hg transport in contaminated soils. J. Hazard. Mater..

[46] Zhou D., Wang D., Cang L., Hao X., Chu L. (2011). Transport and re-entrainment of soil colloids in saturated packed column: Effects of pH and ionic strength. J. Soil Sediment..

[47] Benjamin M.M., Sletten R.S., Bailey R.P., Bennett T. (1996). Sorption and filtration of metals using iron-oxide-coated sand. Water Res..

[48] Kretzschmar R., Barmettler K., Grolimund D., Yan Y.D., Borkovec M., Sticher H. (1997). Experimental determination of colloid deposition rates and collision efficiencies in natural porous media. Water Resour. Res..

[49] Bradford S.A., Simunek J., Bettahar M., Van Genuchten M.T., Yates S.R. (2003). Modeling colloid attachment, straining, and exclusion in saturated porous media. Environ. Sci. Technol..

[50] Adamczyk Z., Siwek B., Zembala M., Belouschek P. (1994). Kinetics of localized adsorption of colloid particles. Adv. Colloid Interface.

[51] Fang J., Shan X., Wen B., Lin J., Owens G. (2009). Stability of titania nanoparticles in soil suspensions and transport in saturated homogeneous soil columns. Environ. Pollut..

[52] Hoek E.M.V., Agarwal G.K. (2006). Extended DLVO interactions between spherical particles and rough surfaces. J. Colloid Interface Sci..

[53] Xu S., Qi J., Chen X.J., Lazouskaya V., Jin Y., Zhuang J. (2016). Coupled effect of extended DLVO and capillary interactions on the retention and transport of colloids through unsaturated porous media. Sci. Total Environ..

[54] Ruckenstein E., Prieve D.C. (1976). Adsorption and desorption of particles and their chromatographic separation. AIChE J..

[55] Bhattacharjee S., Elimelech M. (1997). Surface element integration: A novel technique for evaluation of DLVO interaction between a particle and a flat plate. J. Colloid Interface Sci..

[56] Hamaker H.C. (1937). The London-van der Waals attraction between spherical particles. Physica.

[57] Hogg R., Healy T.W., Fuerstenau D.W. (1966). Mutual coagulation of colloidal dispersions. Trans. Faraday Soc..

[58] Oliveira R. (1997). Understanding adhesion: A means for preventing fouling. Exp. Therm. Fluid Sci..

[59] Lin S., Wiesner M.R. (2012). Deposition of aggregated nanoparticles-A theoretical and experimental study on the effect of aggregation state on the affinity between nanoparticles and a collector surface. Environ. Sci. Technol..

[60] Gomez-Flores A., Bradford S.A., Wu L., Kim H. (2019). Interaction energies for hollow and solid cylinders: Role of aspect ratio and particle orientation. Colloid Surf. Asp..

[61] Sun Y., Gao B., Bradford S.A., Wu L., Chen H., Shi X., Wu J. (2015). Transport, retention, and size perturbation of graphene oxide in saturated porous media: Effects of input concentration and grain size. Water Res..

[62] Zhuang J., Jin Y. (2008). Interactions between viruses and goethite during saturated flow: Effects of solution pH, carbonate, and phosphate. J. Contam. Hydrol..

[63] Bradford S.A., Torkzaban S. (2013). Colloid Interaction Energies for Physically and Chemically Heterogeneous Porous Media. Langmuir.

[64] Shen C.Y., Lazouskaya V., Zhang H.Y., Li B.G., Jin Y., Huang Y. (2013). Influence of surface chemical heterogeneity on attachment and detachment of microparticles. Colloids Surf. Asp..

[65] Zhuang J., Jin Y., Flury M. (2004). Comparison of Hanford colloids and kaolinite transport in porous media. Vadose Zone J..

